# Insecticidal Traits of Variants in a Genotypically Diverse Natural Isolate of Anticarsia Gemmatalis Multiple Nucleopolyhedrovirus (AgMNPV)

**DOI:** 10.3390/v15071526

**Published:** 2023-07-10

**Authors:** Ana Parras-Jurado, Delia Muñoz, Inés Beperet, Trevor Williams, Primitivo Caballero

**Affiliations:** 1 Institute for Multidisciplinary Research in Applied Biology, Universidad Pública de Navarra, 31006 Pamplona, Spain; ana.parras@unavarra.es (A.P.-J.); dmunoz@unavarra.es (D.M.); 2 Departamento de Investigación y Desarrollo, Bioinsectis SL, Polígono Industrial Mocholi Plaza Cein 5, Nave A14, 31110 Noáin, Spain; ines.beperet@bioinsectis.com; 3 Instituto de Ecología AC (INECOL), Xalapa 91073, Veracruz, Mexico; trevor.williams@inecol.mx

**Keywords:** *Baculoviridae*, bioinsecticide, genotypic variant, pathogenicity, virulence, occlusion body production

## Abstract

Outbreaks of *Anticarsia gemmatalis* (Hübner, 1818) (Lepidoptera: Erebidae), a major pest of soybean, can be controlled below economic thresholds with methods that do not involve the application of synthetic insecticides. Formulations based on natural isolates of the Anticarsia gemmatalis multiple nucleopolyhedrovirus (AgMNPV) (*Baculoviridae*: *Alphabaculovirus*) played a significant role in integrated pest management programs in the early 2000s, but a new generation of chemical insecticides and transgenic soybean have displaced AgMNPV-based products over the past decade. However, the marked genotypic variability present among and within alphabaculovirus isolates suggests that highly insecticidal genotypic variants can be isolated and used to reduce virus production costs or overcome isolate-dependent host resistance. This study aimed to select novel variants of AgMNPV with suitable insecticidal traits that could complement the existing AgMNPV active ingredients. Three distinct AgMNPV isolates were compared using their restriction endonuclease profile and in terms of their occlusion body (OB) pathogenicity. One isolate was selected (AgABB51) from which eighteen genotypic variants were plaque purified and characterized in terms of their insecticidal properties. The five most pathogenic variants varied in OB pathogenicity, although none of them was faster-killing or had higher OB production characteristics than the wild-type isolate. We conclude that the AgABB51 wild-type isolates appear to be genotypically structured for fast speed of kill and high OB production, both of which would favor horizontal transmission. Interactions among the component variants are likely to influence this insecticidal phenotype.

## 1. Introduction

The velvetbean caterpillar, *Anticarsia gemmatalis* (Hübner, 1818) (Lepidoptera: Erebidae), is one of the main species of the soybean pest complex. It causes major economic damage to this crop across the Americas [1,2,3,4]. The main strategies employed to control *A. gemmatalis* outbreaks rely on the use of synthetic insecticides, a range of selective or biorational products and the cultivation of transgenic plants expressing Bt insecticidal proteins [5,6,7,8].

The Anticarsia gemmatalis multiple nucleopolyhedrovirus (AgMNPV) (*Baculoviridae*: *Alphabaculovirus*), a natural pathogen of this pest, has played a significant role in integrated pest control programs, and was applied to over ~2 million hectares annually in the early 2000s. The high pathogenicity of the virus results in the control of this pest below economic thresholds following a single application of virus occlusion bodies (OBs) to soybean crops [9]. However, the use of AgMNPV-based insecticides has been drastically reduced over the past decade due to the widespread adoption of transgenic soybean [10,11,12].

One of the key steps in the development of virus-based insecticides is the selection of the active material. This is usually achieved by comparing the insecticidal traits of different natural isolates against a target pest population [13,14]. In nature, nucleopolyhedroviruses exist as heterogeneous populations composed of different genotypes [15,16]. Genotypic variants arise from random molecular events involving recombination, insertion, duplication, and the deletion of genomic sequences or horizontal gene transfer [17,18]. Some of these variations confer distinct insecticidal properties, usually measured in terms of OB pathogenicity metrics, speed of kill, and OB production, which can directly influence virus survival and transmission between susceptible hosts [14,19,20,21,22]. Indeed, nucleopolyhedrovirus isolates comprising variants with divergent insecticidal traits have been identified in several lepidopteran host species, including *Spodoptera exigua* [23], *S. frugiperda* [24,25], and *Helicoverpa armigera* [26].

Furthermore, intraspecific genotypic diversity and phenotypic variation has been described among geographical isolates of a wide array of alphabaculovirus species [14,22,27,28]. Characterization of the genotypic variants present in a wild-type isolate has proved to be important for the selection and development of some of these variants as the active ingredients of virus-based insecticides [29,30,31].

Indeed, the prototype AgMNPV-2D, a majority variant present in AgMNPV formulations against *A. gemmatalis* [32], was originally identified by plaque purification of a wild-type isolate from Brazil [33,34]. However, from field experiences in other host–virus systems [35,36,37], access to a range of highly insecticidal genotypic variants is important in case of isolate-dependent host resistance [38,39,40,41]. Variants can also differ in their OB production traits, which lend some variants to be more amenable than others to mass-production processes necessary for the commercialization of virus insecticides [42].

In this study, we compared several natural isolates of AgMNPV and characterized the genotypic variants present in the most pathogenic isolate, with the aim of comparing the insecticidal traits of the individual variants as potential active substances for novel AgMNPV-based formulations. Specifically, comparative molecular and biological analyses of three AgMNPV isolates resulted in the selection of one of them, from which eighteen genotypic variants were obtained and characterized in terms of OB pathogenicity, speed of kill, and OB productivity.

## 2. Materials and Methods

### 2.1. Insects, Cells, and Virus Isolates

The *Anticarsia gemmatalis* population was established from larvae collected in soybean fields of Tamaulipas, Mexico and maintained at 25 ± 1 °C, 75% relative humidity and 16 h light: 8 h dark photoperiod on a wheatgerm-based semisynthetic diet [43].

Sf9 cells (Gibco, Thermo Fisher Scientific Inc., Waltham, MA, USA) were maintained in Sf-900 II medium (Gibco) at 28 °C.

The Anticarsia gemmatalis multiple nucleopolyhedrovirus (AgMNPV) isolates used in this study were AgMNPV-30WT (hereinafter known as Ag30WT) isolated in Mexico [22], and AgMNPV-ABB15 (AgABB15) and AgMNPV-ABB51 (AgABB51), which both originated from the virus collection of the Institut National de la Recherche Agronomique (INRA), France and were kindly provided by Miguel López-Ferber. These INRA isolates were likely to have been deposited during the studies on AgMNPV by Crozier and Ribeiro [18]. OBs were amplified in fourth instars of *A. gemmatalis* and purified via homogenization of each virus-killed larva in 1 mL of 0.1% sodium dodecyl sulfate (SDS) followed by filtration through muslin and centrifugation at 2400× *g* for 5 min to eliminate insect debris. The resulting pellets were washed and resuspended in 2 vol. milli-Q water. Purified OB suspensions were titrated under phase-contrast microscopy at ×400 using an improved hemocytometer (Paul Marienfeld GmbH, Lauda-Königshofen, Germany) and aliquots of each isolate were stored at −20 °C until required.

Unless otherwise stated, all bioassays and related procedures described in the following sections were performed at 25 °C, 75% relative humidity, 16 h light: 8 h dark photoperiod.

### 2.2. DNA Extraction from OBs

Occlusion derived virions (ODVs) were released by incubating 40 µL of OB suspension (10^10^ OBs/mL) with 100 µL of 0.5 M Na_2_CO_3_ and 360 µL distilled water at 60 °C for 30 min. A supernatant containing the released virions was obtained via centrifugation at 5900× *g* for 5 min and immediately transferred to a new microcentrifuge tube and incubated with 25 µL 10% SDS, 25 µL 0.5 M EDTA, and 15 µL proteinase K (20 mg/mL) at 65 °C for 1 h to degrade the virion membrane and the nucleocapsid and, hence, release the virus genome. Viral DNA was then separated from proteins by adding 150 µL MPC Protein Precipitation Reagent (Epicentre, Illumina Inc., San Diego, CA, USA), vortexing vigorously for 10 s, and pelleting the debris via centrifugation at 10,000× *g* and 4 °C for 10 min. The supernatant was transferred to a new microcentrifuge tube and DNA was pelleted by adding 1 mL ice-cold absolute ethanol and centrifuged at 16,200× *g* at 4 °C for 10 min. The pelleted DNA was washed with 500 µL 70% ethanol, resuspended in 50 µL bidistilled water, and incubated at 60 °C for 15 min.

### 2.3. Cloning of Genotypic Variants

Individual genotypes were obtained from AgABB51 via plaque purification [44]. A concentration of 10^8^ OB/mL was used to inoculate fifth instar *A. gemmatalis* larvae using the droplet feeding method [45]. At 48 h post infection (hpi), infected larvae were bled and hemolymph containing budded virions was collected and stored at −20 °C. Hemolymph was then passed through a 0.45 µm filter and used to prepare six serial dilutions in Sf-900 II (Gibco) medium with antibiotics. A 200 µL volume from each dilution was used to inoculate 5 × 10^5^ Sf9 cells (Gibco) and, at 10 d post infection (dpi), clearly separated individual plaques containing individual clones were collected with a sterile Pasteur pipette and diluted in 300 µL Sf-900 II medium. Clones were then injected into the hemocoel of *A. gemmatalis* fifth instar larvae that were individually placed in the wells of a cell culture plate with a semisynthetic diet and incubated at 25 °C until death or pupation.

### 2.4. Viral DNA Restriction Endonuclease Analysis

For restriction endonuclease analysis, 2 µg viral DNA was digested using two units of HindIII (Fast Digest, Thermofisher, Waltham, MA, USA) for 2 h at 37 °C. DNA fragments were separated via electrophoresis in a 1% agarose gel immersed in TAE buffer (40 mM Tris, 20 mM acetic acid, and 1 mM EDTA pH 8.0) running at 18 V for 15 h. DNA fragments were stained using GelRed (Biotium, Fremont, CA, USA) and photographed on a transilluminator (Gel Doc EZ Imager, Bio-Rad Inc., Hercules, CA, USA).

### 2.5. Biological Activity

The three isolates employed, Ag30WT, AgABB15, and AgABB51, were used to inoculate groups of 28 newly molted second instar larvae of *A. gemmatalis* at three different concentrations (10^3^, 10^5^, and 10^7^ OBs/mL) using the droplet feeding method [45]. Larvae that drank the OB suspensions within 10 min were individualized in wells of a cell culture plate with a piece of semisynthetic diet. Control larvae drank a solution that contained no OBs. These assays were performed in triplicate using different insect batches. Mortality was recorded every 24 h until all larvae were dead or had pupated.

The pathogenicity of AgABB51 OBs, expressed as the median lethal concentration (LC_50_), was determined by the droplet feeding method [45]. Groups of 28 newly molted second instar larvae were allowed to drink OB suspensions of one of the following concentrations, expected to cause between 10% and 90% mortality: 1.2 × 10^3^, 3.7 × 10^3^, 1.1 × 10^4^, 3.3 × 10^4^, and 1.0 × 10^5^ OBs/mL. Control larvae drank a solution that contained no OBs. Larvae that drank the OB suspensions within 10 min were individualized in wells of a cell culture plate with semi-synthetic diet and mortality was recorded at 24-h intervals until all larvae had died or pupated. This assay was performed in triplicate with different insect batches. Concentration–mortality data were analyzed via Probit regression to estimate LC_50_ values using the software POLO Plus (Leora) [46].

Median time to death (MTD) was estimated in AgABB51-infected second instars that consumed a suspension of 6.8 × 10^4^ OBs/mL (estimated to result in ~90% mortality). Larvae that drank the inoculum within 10 min were individualized with a semisynthetic diet and mortality was recorded every 8 h until all larvae had died or pupated. Control larvae consumed a solution without OBs. The study was performed on three batches of insects. In order to estimate MTD values, a survival analysis was performed using the ‘Survival’ package [47] in R (v4.2.2) [48]. The Akaike Information Criterion (AIC) was calculated in order to identify the best fitting model.

OB production was determined in fifth instars inoculated with an LC_99_ (10^8^ OBs/mL) concentration of AgABB51 OBs using the droplet feeding method [45]. In each of the three biological replicates, dead larvae were collected individually in a 1.5 mL microcentrifuge tube and OBs were purified in a total volume of 1 mL with milli-Q water. OB suspensions from 26 larvae from each replicate were titered in a Neubauer hemocytometer. Data were subjected to a Shapiro–Wilk normality test and analysis of variance (ANOVA) followed by a post hoc Tukey’s HSD test in R (v4.2.2) [48].

An inoculum concentration equal to the LC_50_ of AgABB51 OBs (1.1 × 10^4^ OBs/mL) was used to evaluate the mortality response of *A. gemmatalis* second instar larvae to each of the genotypic variants isolated in Section 2.3. Larvae were inoculated using the droplet feeding method [45] and incubated individually on a semisynthetic diet in the wells of a cell culture plate. Mortality was registered daily until all individuals were dead or pupated. The bioassay was performed on three batches of insects with appropriate controls. Percentage of mortality values were analyzed using one-way ANOVA followed by a post hoc Tukey’s HSD test in SPSS v25.0 software [49].

### 2.6. Genotypic Variant Selection and Biological Characterization

The genotypic variants that induced higher mortality responses than the wild-type isolate (AgABB51) were selected for further characterization in terms of LC_50_, MTD, and OB production as described in Section 2.5, whereas MTD values were subjected to a log-rank test using the package ‘Survival’ [47] and a post hoc Bonferroni-corrected pairwise *t*-test. OB production data were subjected to a Shapiro–Wilk normality test and one-way ANOVA followed by a post hoc Tukey’s HSD test. All analyses were performed using R (v4.2.2) [48].

## 3. Results

### 3.1. Identification of AgMNPV Isolates Using Restriction Endonuclease Analysis

**Figure 1:**
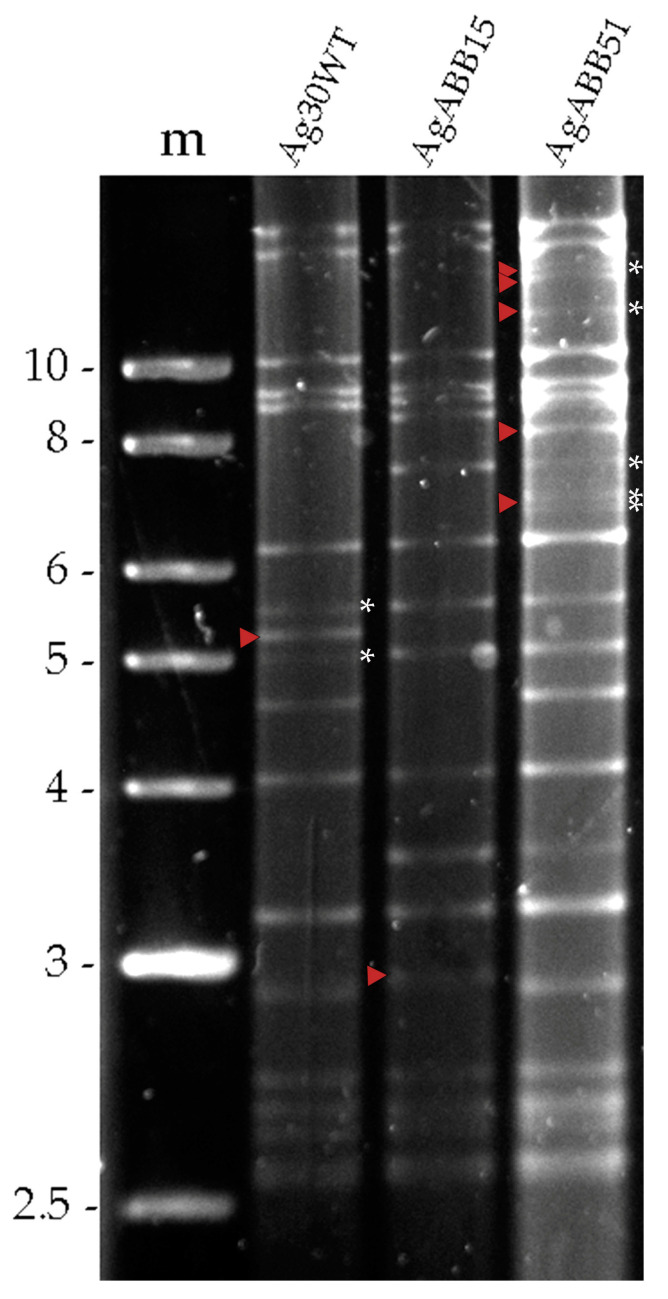
Restriction endonuclease profiles of the genomic DNA of three different AgMNPV isolates following treatment with HindIII. m denotes the molecular marker. Fragment size in kilobases (Kb) is shown on the left. Red arrowheads indicate restriction fragment length polymorphisms (RFLPs) and asterisks on the left of each lane indicate the presence of submolar bands.

Analysis of the genomic DNA of three different AgMNPV isolates (Ag30WT, AgABB15 and AgABB51) revealed three slightly different REN profiles, as judged by the distinct restriction fragment length polymorphisms (Figure 1). All the isolates appeared to generate restriction fragments in submolar concentrations, indicating the presence of different genotypic variants within each isolate (Figure 1).

### 3.2. Mortality Response to AgMNPV Isolates

Similar mortality values were observed at the lowest and highest viral concentrations used for the three candidate isolates, but isolate AgABB51 resulted in higher larval mortality at 10^5^ OBs/mL (Table 1). Thus, we selected AgABB51 for further characterization.

### 3.3. Biological and Genotypic Characterization of AgABB51

The pathogenicity of AgABB51 OBs, in terms of the LC_50_, was estimated at 1.1 × 10^4^ OBs/mL. A median time to death value of 138.6 h post inoculum (hpi) was estimated through a survival analysis using the log-normal model, which was identified as the best fitting model via comparison of distribution-dependent AIC values. The mean OB yield was 1.23 × 10^9^ OBs in *A. gemmatalis* fifth instars (Table 2).

**Figure 2:**
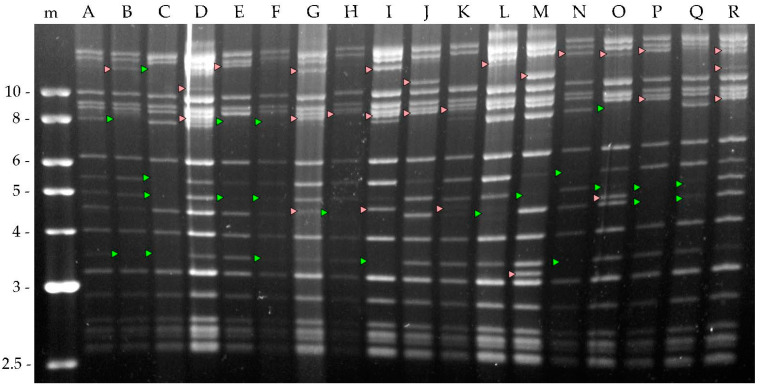
HindIII restriction endonuclease profiles of the genomic DNA of each of the 18 different AgABB51 genotypic variants (A to R). m denotes the molecular marker. Fragment size in kilobases (Kb) is shown on the left. The profile of genotype A is used as a reference to identify the presence (red arrowheads) or absence (green arrowheads) of characteristic restriction fragments in the other genotypic variants. Genotype A was selected for this purpose because of its similarity to the wild-type isolate (AgABB51) (shown in Figure 1).

A total of 128 different clones were obtained from the plaque assay and were each amplified by injection into *A. gemmatalis* fifth instar larvae. OBs from virus-killed larvae were collected and the viral DNA were digested using HindIII to determine differences in their genomic profile. Eighteen different genotypic variants were obtained and named A to R (Figure 2).

**Figure 3:**
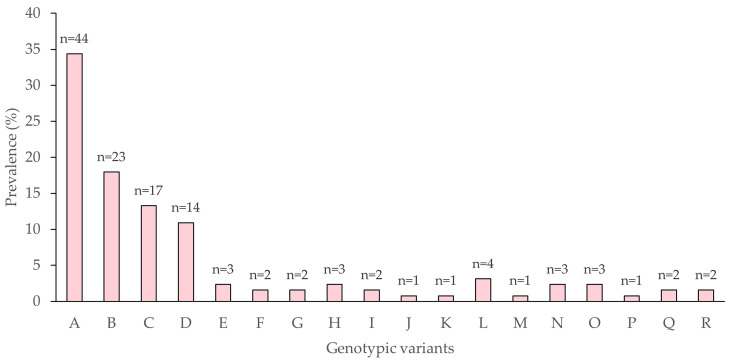
Frequency of AgABB51 genotypic variants A through R. The *n*-value above each column indicates the number of clones exhibiting each variant’s restriction profile out of a total of 128 clones.

The frequency of each variant was determined as the number of times each characteristic restriction profile was observed in the 128 clones (Figure 3).

### 3.4. Biological Characterization of AgABB51 Genotypic Variants

**Figure 4:**
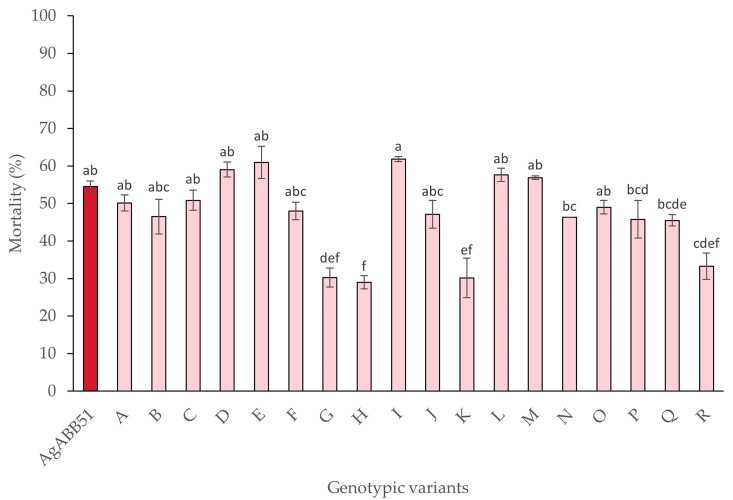
Mean percentage of mortality caused by AgABB51 wild-type isolate and each of the genotypic variants A–R on *A. gemmatalis* second instars inoculated with 1.1 × 10^4^ OBs/mL. Error bars indicate the standard error. Different lowercase letters indicate significant differences between variants (ANOVA, Tukey HSD; *p* < 0.05).

The mean percentage of mortality in larvae inoculated with each of the different genotypic variants and the wild-type AgABB51 varied significantly (F_18,38_ = 12.64; *p* < 0.001) (Figure 4). However, larvae inoculated with genotypic variants A, B, C, D, E, F, J, I, L, M, N, O, P, and Q showed mortalities as high as those inoculated with AgABB51 OBs in the initial bioassays, at the concentration equal to the LC_50_ of AgABB51 (1.1 × 10^4^ OBs/mL). Five of these variants were selected for further characterization based on the high mortality values in this preliminary assay (Table 3).

The pathogenicity of AgABB51 and the five selected genotypic variants ranged from 1 × 10^4^ OBs/mL (AgABB51) to 7.6 × 10^3^ OBs/mL (variant I); there were no significant differences detected among these values (Table 3).

**Figure 5:**
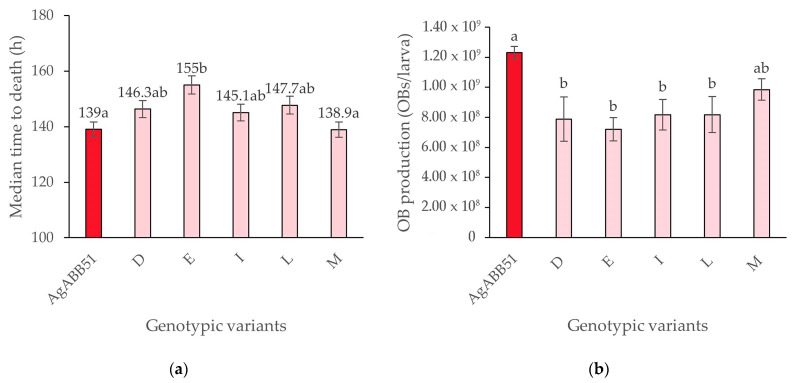
(**a**) Median time to death (MTD) values for the AgABB51 isolate and selected genotypic variants in *A. gemmatalis* second instars. Error bars indicate standard error and different lowercase letters indicate significant differences between variants (Bonferroni-adjusted *t*-test; *p* < 0.05). (**b**) OB production values obtained for AgABB51 and the selected genotypic variants in *A. gemmatalis* fifth instars. Error bars indicate standard error and different lowercase letters indicate significant differences between variants (Tukey HSD test; *p* < 0.05).

Survival analysis using the log-normal model revealed MTD values that varied between 155.0 h (variant E) and 138.9 h (variant M), compared to 139.0 h for the reference AgABB51 isolate (log-rank χ^2^ = 16.3, d.f. = 5, *p* < 0.05). However, a post hoc Bonferroni analysis revealed that only the MTD values of AgABB51 and variant M differed significantly compared to variant E, whereas the other variants showed intermediate MTD values (Figure 5a).

In terms of OB production, none of the selected genotypic variants were more productive than the AgABB51 isolate and larvae infected by the variants D, E, I, and L produced fewer OBs than those infected by the AgABB51 isolate (F_5,121_ = 8.533; *p* < 0.001) (Figure 5b).

## 4. Discussion

Three field-collected AgMNPV isolates were compared in terms of restriction endonuclease profile characteristics and their respective insecticidal properties. AgABB51 was recognized as the most genotypically diverse, as judged by the greater number of RFLPs and submolar fragments within its restriction profile, suggesting the presence of more than one genotypic variant in the wild-type isolate. The *A. gemmatalis* mortality response induced by these isolates was tested at three different concentrations. Similar mortality values were observed at the lowest and highest viral concentrations, although AgABB51 resulted in the highest mortality response (87%) at an intermediate inoculum concentration (Table 1). AgABB51 was, therefore, selected for further biological and genotypic characterization. The OB pathogenicity (LC_50_) and speed-of-kill (MTD) values of this isolate were 1.1 × 10^4^ OBs/mL and 138.6 h in second instars, respectively, whereas OB production averaged 1.23 × 10^9^ OBs in each virus-killed fifth instar (Table 2). Although the AgMNPV-2D variant was not included in our bioassays and comparisons of the results of different experimental events tend to be difficult, the AgABB51 LC_50_ and MTD values seemed lower than those of AgMNPV-2D, suggesting that AgABB51 might possess traits that would favor its use as a biological insecticide [22,32,50,51].

A total of 128 clones were obtained via plaque purification of the AgABB51 isolate. From these, 18 different genotypic variants (AgABB51-A to R) were identified based on their HindIII restriction profiles (Figure 2). A similar heterogeneity has been reported in the population structure of other alphabaculoviruses [21,52,53], including other AgMNPV isolates [18,22,34,54].

Minor variation in the genome sequence can have a direct impact on the insecticidal phenotype of genotypic variants [42,55,56]. In line with this idea, four of the AgABB51 variants caused a mortality lower than that of AgABB51-wt, whereas 14 variants caused similar mortality to that of the wild-type isolate, including the five selected variants D, E, I, L, and M.

There was no clear correlation between the frequency of the variant in the isolate (Figure 3) and virus-induced mortality (Figure 4), as previously observed by other authors [21,22,56,57]. One reason may be that variants that are amenable to cell culture conditions are not necessarily the most pathogenic or transmissible variants in nature [27,57,58,59]. The alternative in vivo cloning method was originally developed to purify variants from alphabaculovirus isolates [60,61], but is a highly labor-intensive method that has largely been abandoned in favor of plaque purification [21].

The five selected variants from AgABB51 were not significantly more pathogenic than the AgABB51-wt (Table 3). None of these variants displayed a faster speed of kill or higher OB production than AgABB51-wt (Figure 4). Indeed, for most of these variants slower speed of kill was associated with lower OB production per larva, which deviates from the finding that speed of kill is often negatively correlated with OB production, presumably because the virus has more time to replicate and the insect can continue to grow during the infection period [62,63,64]. It appears, therefore, that the AgABB51 isolate is genotypically structured so that the speed of kill favors rapid insect-to-insect transmission in combination with high OB production which also increases the probability of horizontal transmission. These emergent traits likely arise from interactions among the component genotypes, a phenomenon also observed in experiments involving the production of variant mixtures in other alphabaculoviruses [65].

On a more general note, the study of the highly diverse AgABB51 population also highlights the importance of collective infectious units in virus transmission [15,66,67]. The polyploid nature of viruses that disperse in groups, such as alphabaculoviruses, has important consequences for viral evolution, as it increases the probability of coinfection, recombination, and complementation among coinfecting variants. Cells infected with multiple virus genomes will favor interactions between viruses, that may result in changes in viral pathogenesis, the diversity of the virus progeny, and the evolution of host resistance [67].

## 5. Conclusions

Plaque purification and restriction endonuclease analysis revealed that a natural isolate of AgMNPV, named AgABB51, comprised 18 genotypically distinct variants that differed in OB pathogenicity. Five variants that elicited a high mortality response were selected for further characterization. The lethal concentration metrics and speed of kill of the variants were generally similar to that of the natural isolate, whereas OB production was generally lower than observed in the natural isolate. Future studies should examine the role of interactions among the component genotypes that are likely to affect the insecticidal phenotype of the virus and its efficacy as a biological pesticide.

## Figures and Tables

**Table 1:** Percentage of mortality (± SE) observed for each AgMNPV isolate in *A. gemmatalis* second instars. Values in parentheses indicate the numbers of larvae tested at each inoculum concentration.

Virus	10^3^ OBs/mL	10^5^ OBs/mL	10^7^ OBs/mL
Ag30WT	8 ± 4 (78)	58 ± 8 (73)	98 ± 2 (83)
AgABB15	11 ± 5 (78)	72 ± 8 (81)	100 ± 0 (79)
AgABB51	10 ± 6 (82)	87 ± 1 (77)	100 ± 0 (74)

None of the control insects (*n* = 72–78 larvae per isolate) died of polyhedrosis disease.

**Table 2:** Estimated median lethal concentration (LC_50_) and median time to death (MTD) values for AgABB51 in *A. gemmatalis* second instars and OB production values for AgABB51 in *A. gemmatalis* fifth instars.

Virus	LC_50_ * (OBs/mL)	95% Confidence Limits		MTD (h)	95% Confidence Limits		OB Production (OBs/Larva)	95% Confidence Limits	
		Low	High		Low	High		Low	High
AgABB51	1.1 × 10^4^	8.5 × 10^3^	1.5 × 10^4^	138.6	134.3	143.0	1.23 × 10^9^	1.15 × 10^9^	1.31 × 10^9^

* LC_50_ estimated using Probit regression with a slope (± SE) 1.634 ± 0.148 and intercept (± SE) −6.612 ± 0.612 (goodness-of-fit test χ^2^ = 0.2198, d.f. = 3, *p* = 0.974, heterogeneity = 0.0733).

**Table 3:** Lethal concentration estimates and relative potency values for AgABB51 and selected individual genotypic variants in *A. gemmatalis* second instars.

Variant	LC_50_ (OBs/mL)	Relative Potency	95% Confidence Limits		LC_90_ (OBs/mL)	Relative Potency	95% Confidence Limits		Slope (±SE)
			Low	High			Low	High	
AgABB51	1.0 × 10^4^	1	-	-	6.9 × 10^4^	1	-	-	1.54 ± 0.128
D	8.8 × 10^3^	1.15	0.84	1.56	5.3 × 10^4^	1.30	0.77	2.11	1.64 ± 0.138
E	8.0 × 10^3^	1.26	0.92	1.72	4.4 × 10^4^	1.55	0.91	2.65	1.72 ± 0.147
I	7.6 × 10^3^	1.33	0.97	1.81	4.1 × 10^4^	1.68	1.00	2.9	1.75 ± 0.152
L	9.2 × 10^3^	1.10	0.80	1.50	5.6 × 10^4^	1.21	0.70	2.13	1.62 ± 0.141
M	9.2 × 10^3^	1.10	0.74	1.36	4.9 × 10^4^	1.40	0.83	2.35	1.65 ± 0.135

Regressions did not differ significantly as hypotheses for parallelism (χ^2^ = 2.67, df = 5, *p* > 0.05) and equality (χ^2^ = 7.83, df = 10, *p* > 0.05) were not rejected. The relative potencies were calculated as the ratio of effective concentrations relative to the wild-type AgABB51.

## Data Availability

The data presented in this study are available on request from the corresponding author.
